# Pneumococcal vaccination rates in immunocompromised patients in Germany: A retrospective cohort study to assess sequential vaccination rates and changes over time

**DOI:** 10.1371/journal.pone.0265433

**Published:** 2022-03-22

**Authors:** Ralf Sprenger, Dennis Häckl, Nils Kossack, Julia Schiffner-Rohe, Jessica Wohlleben, Christof von Eiff

**Affiliations:** 1 Pfizer Pharma GmbH, Berlin, Germany; 2 Scientific Institute for Health Economics andfig Health System Research (WIG2 GmbH), Leipzig, Germany; 3 University Leipzig, Leipzig, Germany; University of Florida, UNITED STATES

## Abstract

**Background:**

Pneumococcal vaccination is recommended by the German Standing Committee on Vaccination (STIKO) for infants, elderly 60+ years and patients at risk. In 2016, a sequential pneumococcal vaccination schedule (conjugate vaccine followed by polysaccharide vaccine 6–12 months later) supplemented this recommendation for immunocompromised patients ≥2 years of age. Previous research showed low pneumococcal vaccination rates (pnc-VR) in this vulnerable group. Moreover, no evidence is available on adherence to the newer sequential schedule. This study aimed to analyze the development of pnc-VRs in immunocompromised patients and rates of sequential vaccinations according to the STIKO recommendations.

**Methods:**

Using a representative health claims database, we assigned incident immunocompromised patients ≥2 years of age to one of two successive cohorts to observe trends over time: cohort A (first diagnosis of immunocompromised condition between 01/2013 and 12/2014), and cohort B (first diagnosis between 01/ 2015 and 12/2017). Pnc-VR within two years after first diagnosis and cumulative pnc-VR was compared among both cohorts. In cohort B, we assessed sequential pnc-VR within 15 months of the first vaccination. For additional analyses, patients were stratified by age, gender and immunocompromising condition.

**Results:**

Cohort A and B comprised 193,521 and 289,279 patients, respectively. Overall pnc-VR increased over time from 4.3% (cohort A; 95%-confidence interval: 4.3%-4.4%) to 6.0% (cohort B; 5.9%-6.1%), with highest pnc-VRs in men ≥60 years (11.3%: 11.1%-11.6%) and HIV patients (15.2%: 13.1%-17.4%). Cumulative pnc-VRs in cohort B were higher in any quarter following diagnosis when compared with cohort A. Overall sequential pnc-VR in cohort B was 4.0% (3.7%-4.3%), with a higher rate observed in patients aged 16–59 (6.8%: 6.0%-7.7%) vs. patients aged ≥60 years (3.1%: 2.8%-3.4%).

**Conclusion:**

While some improvements were seen over time, pnc-VRs remain very low in immunocompromised patients, as did sequential vaccination rates. Current recommendations to protect immunocompromised patients from pneumococcal infections are not being sufficiently implemented.

## Background

*S*. *pneumoniae* is estimated to cause approximately 30%-50% of community-acquired pneumonia (CAP) requiring hospitalization in adults in Europe and the United States (US) [1, 2]. The Institute for Quality Assurance and Transparency in Healthcare (IQTIG) estimates there to be approximately 255,000 CAP cases annually in Germany (non-hospital acquired and in patients over 18 years) [3]. A study based on claims data recently published by Theilacker et al. [4] also indicated high CAP incidence rates among adults in Germany; the authors reported an overall incidence rate of 1,054 cases per 100,000 person years of observation for the calendar year 2015. This study also revealed high mortality rates; 18.5% of patients ≥18 years hospitalized with CAP died during their inpatient stay, 22.9% died within 30 days and 44.5% died within one year following CAP onset [4]. CAP also has an impact on healthcare resources; Campling et al. [5] found significantly higher healthcare resource utilization in patients with selected underlying comorbidities after hospitalization forCAP when compared to matched patients without CAP. Apart from higher resource utilisation and costs, this study found higher odds ratios of hospital-acquired pneumonia in patients suffering from one of six investigated comorbidities, when compared with patients admitted to hospital for tooth extraction, with odds ratios of 1.18 (95%-CI 1.13–1.23) in patients with diabetes, and to 5.48 (5.28–5.70) in patients with chronic respiratory disease [6].

Pelton et al. [7] found that patients with ≥2 chronic diseases had a comparable or even higher risk of all-cause pneumonia than immunocompromised patients in the same age group, and compared with their healthy controls, immunocompromised adults were 3.2 to 4.1 times more likely to develop pneumonia. Finally, in a secondary analysis of a global multicenter study on adult patients, 17.6% of CAP patients were found to have one or more risk factors for being immunocompromised, with chronic use of systemic steroids (used in immunosuppressive therapy) being the most frequent risk factor [8].

Due to the increased risk of pneumococcal disease (PD) in vulnerable populations, the German Standing Committee on Vaccination (STIKO, Ständige Impfkommission) recommends pneumococcal vaccination as routine for infants (using a 2+1 schedule with immunisations in the 2^nd^, 4^th^ and 11^th^ months of life, with either PCV10 or PCV13, both conjugated pneumococcal vaccines) and for seniors ≥60 years (with the polysaccharide vaccine PPSV23, with potential repeated vaccinations after an interval of at least 6 years). A sequential schedule is recommended (PCV13 followed by PPSV23 after an interval of 6–12 months) for patients with defined underlying diseases (patients with inherited or acquired immunodeficiency or immunosuppression or patients < 16 years with defined chronic diseases or patients with anatomic or foreign body-associated risks for a pneumococcal meningitis). However, only a single PPSV23 immunisation is recommended in patients ≥16 years with particular chronic diseases [9]. Without a national registry to monitor vaccination rates, reports of current rates from sample populations provide the best information available for this purpose. For the first quarter of 2020, the Robert Koch Institute (RKI) reported a 19% pneumococcal vaccination rate in adults (≥18 years) with a vaccination indication due to a pre-existing condition [10]. In a claims data-based cohort study, Braeter et al. [11] found that 10.2% of statutory health insurance (SHI)-insured individuals aged 60–64 years had received a pneumococcal vaccine within a 5-year follow-up period. Of all patients with a chronic disease, 15% had received the vaccine. Schmedt et al. [12] used German healthcare claims data to analyze vaccination rates in patients who were newly diagnosed (i.e. incident) as immunocompromised in the years 2013 and 2014, and found vaccination rates of only 4.4% (95%-CI 4.3%-4.5%) within two years following the incident diagnosis of the condition [9].

The present study aimed to provide a follow-up to the study by Schmedt et al. [12], describing the development of vaccination rates in immunocompromised patients over time, and further considering rates of the sequential vaccination recommended by STIKO in August 2016.

## Methods

### Data source

The study used a sample from the WIG2 (*Wissenschaftliches Institut für Gesundheitsökonomie und Gesundheitssystemforschung*, the Scientific Institute for Health Economics and Health System Research) database—a healthcare claims database with longitudinal data from more than 4 million patients in Germany and at the time of analysis seven German SHIs. Claims data are transferred from SHI data centers to the database. All patient-level data in the database is anonymized and only aggregated data (n≥5) is reported according to German data protection regulations, with no independent ethics committee approval needed. The database provides a representative sample (in terms of age, gender, and morbidity) of the German population and was benchmarked against the total German SHI population [13]. We used data from January 1^st^, 2011 to December 31^st^, 2019 (including baseline and follow-up periods) for analyses.

The WIG2 database includes demographic data (age, gender, residential region), data on outpatient care (diagnoses, procedures, physician specialty, costs), inpatient care (length of stay, procedures, main and secondary diagnoses and reasons for admission and discharge), pharmaceutical data (drugs and quantity dispensed by Anatomical Therapeutic Chemical classification (ATC) codes, and prescribing physician specialty), and finally information on medical devices and allied health services (therapy and duration). For this study, information on diseases and vaccines were drawn from ICD-10 GM (International Classification of Disease, version 10 German modification), OPS (*Operationen- und Prozedurenschlüssel*, German classification of procedures), EBM (*Einheitlicher Bewertungsmaßstab*, German physician fee schedule), or ATC codes.

Pneumococcal vaccination was identified by the vaccine documentation number (EBM code). Different codes are documented for each routine infant vaccination (˂2 years), routine vaccination for patients aged ≥60 years or recommended vaccination due to immunocompromising condition or chronic disease, however they do not differentiate between the particular vaccine administered (PCV13 or PPSV23) (Table 1).

**Table 1:** Documentation codes for Pneumococcal vaccination (G-BA, 2020) [14].

Vaccination	First dose of vaccine schedule	Booster dose
Routine vaccination for infants 0–24 months	89118	89118
Routine vaccination for individuals aged ≥60 years	89119	89119 R*
Recommended for immunocompromised (congenital or acquired) individuals, for chronic disease	89120**	89120 R**

* no routine booster vaccination, according to the German Federal Joint Committee (Gemeinsamer Bundesausschuss, G-BA)

**For sequential vaccinations, the vaccine code 89120 was used for both the PCV13 and the PPSV23 doses.

### Study design

Vaccination rates of incident immunocompromised patients were assessed and compared among two cohorts. Entry into cohorts depended on the year of diagnosis of the incident immunocompromising condition (cohort A: two-year index period with incident diagnosis between January 2013 and December 2014; cohort B: three-year index period with incident diagnosis between January 2015 and December 2017), with a two-year baseline period used for each patient to ensure that the diagnosis was incident.

We applied the same methods as Schmedt et al. [12] to both cohorts, allowing us to better compare results. While the methodology was the same, we referred to our study population as immunocompromised patients, whereas the Schmedt et al. [12] study used the term high-risk to refer to their population with the same criteria. Immunocompromised patients (as outlined by STIKO) included those with functional or anatomical asplenia, sickle cell diseases and other hemoglobinopathies, malignant neoplasms (excluding non-melanoma skin cancer), stem cell transplantation, HIV infection, chronic renal failure, chronic severe liver diseases, use of immunosuppressants (e.g. due to autoimmune disease like rheumatoid arthritis or organ transplantation), and other immunodeficiencies (such as diseases of white blood cells) (see S1 Table).

The date of the first documentation of an immunocompromising condition was considered as the index date on which the patient entered the study. Each patient was followed for two years to evaluate pneumococcal vaccinations.

The index date (incident diagnosis) was the admission date for inpatient diagnoses, and since outpatient diagnoses are documented only by quarter, the date of the first EBM code reimbursed by the diagnosing physician was considered as index date. Since some indications were identified by OPS and EBM codes for specific treatments (for example dialysis), the exact date documented was used as the index date.

### Study population

The study population fulfilled all of the following inclusion criteria: (1) at least one documented incident immunocompromised condition diagnosis during study entry period for the cohort, and none in the two year-baseline period prior; (2) continuous insurance of at least two years prior to the first diagnosis of the immunocompromised condition (baseline period); (3) continuous insurance until December 31^st^, 2016 (cohort A) or December 31^st^, 2019 (cohort B), or until death; and (4) at least two years of age at index date.

Patients were excluded from the study population if, during the baseline period, they had either (1) at least one documented diagnosis code (indicating patient was immunocompromised), or (2) a claim for a pneumococcal vaccination (Table 1).

### Data analysis

#### Subgroup analyses

We calculated vaccination rates in disease-specific subgroups by the immunocompromised condition documented (see Table 3). In another subgroup analysis we evaluated vaccination rates by specialty of the physician that first diagnosed the immunocompromising condition, and by the physician specialty that administered the first vaccination in both cohorts.

#### Cumulative vaccination rate

We estimated cumulative vaccination rates by analysing the vaccination rates after the first diagnosis of the immunocompromised condition by quarter (over eight quarters), with corresponding 95% CIs and reported per cohort. We used this analysis to consider how much time elapsed from diagnosis to vaccination, assuming vaccination rates would be higher soon after the diagnosis, as recommended by STIKO [9].

#### Sequential pneumococcal vaccination

Since the sequential vaccination was first added to the STIKO recommendations in August 2016 (PCV13 followed by a PPSV23 vaccine 6–12 months later), we did this analysis for cohort B only [15]. The sequential vaccination rate in immunocompromised patients was calculated as the ratio of patients receiving a second pneumococcal vaccination within a time period of up to 15 months after first vaccination, among all patients who received a first vaccination. As no documentation code for sequential vaccination is available, we used the documentation codes 89119 and 89120. To account for a limited follow-up (as only two years of follow-up data from the diagnosis date were available), a cumulative incidence analysis of sequential vaccination distributed over time was chosen. Furthermore, we evaluated the time in days from the first to the second vaccination, reported overall and stratified by age groups (16–59, 60+ years); 95%-CIs were reported as well (see S4 Table).

All analyses were conducted using R 3.6.

## Results

### Pneumococcal vaccination rate

The pneumococcal vaccination rate was defined as the proportion of patients who received a pneumococcal vaccination (using all documentation numbers in Table 1 to avoid any impact of incorrect coding) within two years after the diagnosis of an incident immunocompromising condition (i.e. index date) in the study population. We calculated 95% confidence intervals (CI), assuming a binomial distribution. Vaccination rates were reported overall and stratified by age group (2–15, 16–59, and 60+ years), gender, and region (Eastern German states vs. Western German states, see S2 Table). Furthermore, we stratified the pneumococcal vaccination rate by presence of a chronic disease condition during baseline. Chronic diseases considered were chronic heart disease, chronic pulmonary disease (including asthma), diabetes treated with oral antidiabetics or insulin and neurological disorders (Table 2).

**Table 2:** Immunocompromised patient characteristics with a first diagnosis of a condition for which pneumococcal vaccination is recommended as per STIKO guidelines.

		Cohort A	Cohort B
**Gender and age group distribution n, (%)**	Total number of subjects	193,521 (100.0%)	289,279 (100.0%)
	Male	86,349 (44.6%)	132,710 (45.9%)
	Female	107,172 (55.4%)	156,569 (54.1%)
	Age 2–15	10,225 (5.3%)	12,053 (4.2%)
	Age 16–59	113,955 (58.9%)	157,980 (54.6%)
	Age ≥60	69,341 (35.8%)	119,246 (41.2%)
**Conditions (congenital or acquired) resulting in immunodeficiency at index date n, (%)** *	Functional or anatomic asplenia sickle cell diseases and other hemoglobinopathies	1,966 (1.0%)	2,966 (1.0%)
	Other immunodeficiency	105,977 (54.8%)	148,717 (51.4%)
	Malignant neoplasms excluding non-melanoma skin cancer	39,619 (20.5%)	60,070 (20.8%)
	Stem cell transplantation	10 (0.0%)	18 (0.0%)
	HIV infection	832 (0.4%)	1,040 (0.4%)
	Chronic renal failure	35,644 (18.4%)	64,701 (22.4%)
	Chronic severe liver disease	7,311 (3.8%)	10,654 (3.7%)
	Immunosuppressant use	7,498 (3.9%)	10,831 (3.7%)
**Chronic disease conditions n, (%)** *	No relevant chronic disease	91,853 (47.5%)	129,140 (44.6%)
	Patient has a relevant chronic disease	101,668 (52.5%)	160,139 (55.4%)
	Chronic heart disease	45,450 (23.5%)	77,135 (26.7%)
	Chronic pulmonary disease	57,545 (29.7%)	88,131 (30.5%)
	Diabetes treated with oral antidiabetics or insulin	21,017 (10.9%)	36,527 (12.6%)
	Neurological disorders	24,445 (12.6%)	43,391 (15.0%)

*The same patient may appear in several different subgroups.

### Study population

**Fig 1:**
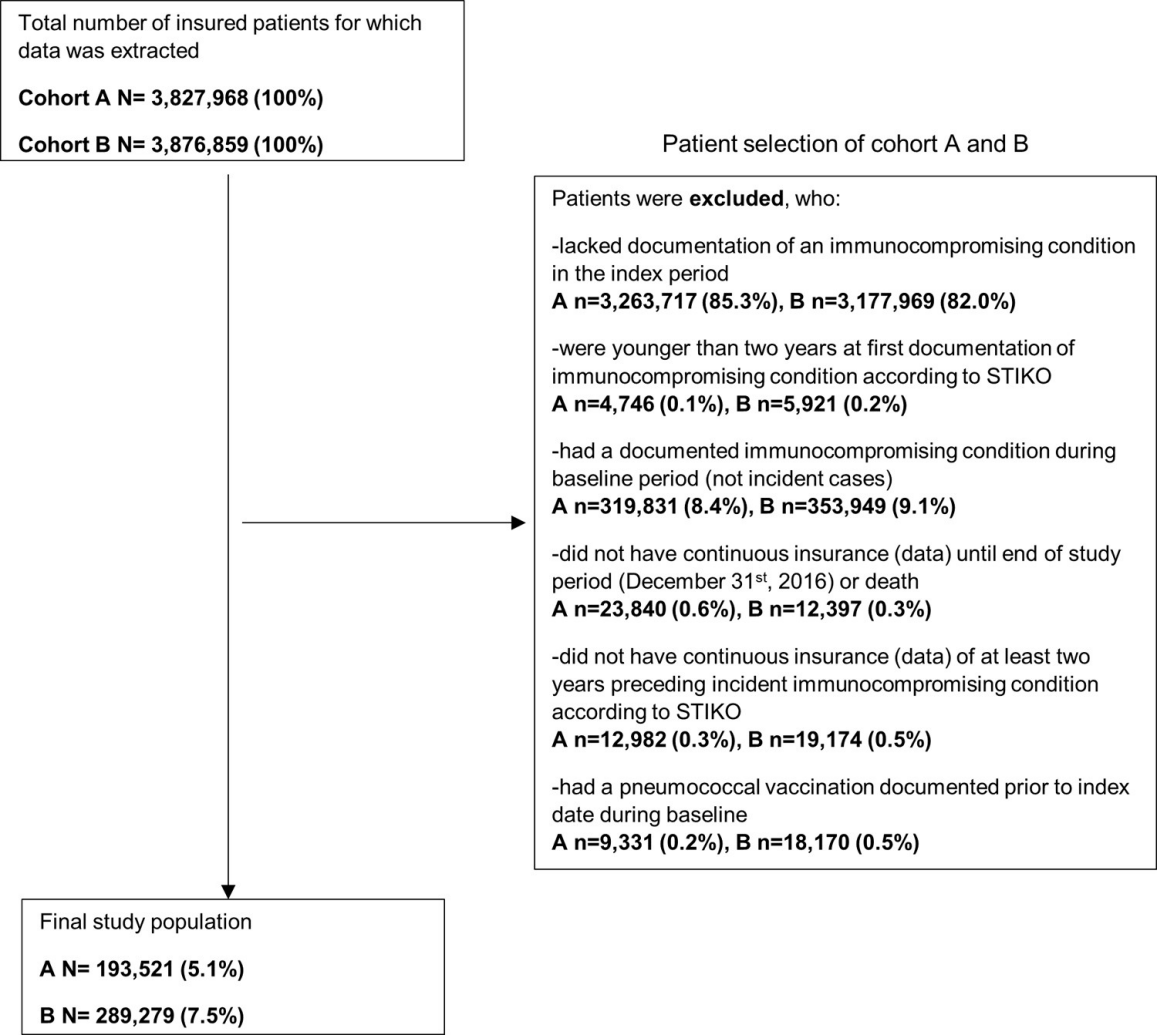
Selection of patient populations for cohorts A and B.

From 3,827,968 in the database, 193,521 (5.1%) patients were eligible to be included in cohort A (Fig 1) over the two-year index period; 54.4% were female, and 58.9% were between 16 and 59 years old.

From 3,876,859 in the database used to examine cohort B 289,279 (7.5%) patients were eligible in the three-year index period, i.e. one year more than for cohort A, among which 54.1% were female and 54.6% were between the ages of 16 and 59 years. The three most frequently observed conditions in immunocompromised patients entering the cohort (by ICD-10 GM code) were other immunodeficiencies, malignant neoplasms excluding non-melanoma skin cancer, and chronic renal failure (see Table 2). More than half of the immunocompromised patients in both cohorts also had at least one underlying chronic disease during baseline, for which STIKO also recommends pneumococcal vaccination (Table 2).

### Pneumococcal vaccination rates within two years of diagnosis of immunocompromised condition

In both cohorts, males in all age groups had higher vaccination rates than females in the same age group. Males aged ≥60 years had the highest vaccination rates (9.0%; 8.7%-9.3% (A) and 11.3%; 11.1%-11.6% (B)) (Table 3). Vaccination rates increased in nearly all groups between cohort A and B, with the largest increase seen in patients aged ≥60 years.

**Table 3:** Pneumococcal vaccination rates with 95% CI within two years following diagnosis of immunocompromised condition.

	Cohort A: Overall			Cohort B: Overall		
	N cohort	N vaccinated	Vaccination rates (95% CI)	N cohort	N vaccinated	Vaccination rates (95% CI)
**Overall**	193,521	8,401	4.3% (4.3%-4.4%)	289,279	17,354	6.0% (5.9%-6.1%)
**Females**	107,172	3,963	3.7% (3.6%-3.8%)	156,569	8,148	5.2% (5.1%-5.3%)
**Males**	86,349	4,438	5.1% (5.0%-5.3%)	132,710	9,206	6.9% (6.8%-7.1%)
**Age 2–15**	10,225	72	0.7% (0.6%-0.9%)	12,053	103	0.9% (0.7%-1.0%)
**Age 16–59**	113,955	2,331	2.0% (2.0%-2.1%)	157,980	3,971	2.5% (2.4%-2.6%)
**Age ≥60**	69,341	5,998	8.7% (8.4%-8.9%)	119,246	13,280	11.1% (11.0%-11.3%)
**Female age 2–15**	5,152	34	0.7% (0.5%-0.9%)	6,140	43	0.7% (0.5%-0.9%)
**Female age 16–59**	68,061	1,105	1.6% (1.5%-1.7%)	92,403	1,753	1.9% (1.8%-2.0%)
**Female age ≥60**	33,959	2,824	8.3% (8.0%-8.6%)	58,026	6,352	10.9% (10.7%-11.2%)
**Male age 2–15**	5,073	38	0.7% (0.5%-1.0%)	5,913	60	1.0% (0.8%-1.3%)
**Male age 16–59**	45,894	1,226	2.7% (2.5%-2.8%)	65,577	2,218	3.4% (3.2%-3.5%)
**Male age ≥60**	35,382	3,174	9.0% (8.7%-9.3%)	61,220	6,928	11.3% (11.1%-11.6%)
**Immunocompromising condition **						
**Functional or anatomic asplenia sickle cell diseases and other hemoglobinopathies**	1,966	142	7.2% (6.1%-8.4%)	2,966	214	7.2% (6.3%-8.2%)
**Other immunodeficiency**	105,977	3,374	3.2% (3.1%-3.3%)	148,717	6,373	4.3% (4.2%-4.4%)
**Malignant neoplasms excl. non-melanoma skin cancer**	39,619	1,953	4.9% (4.7%-5.1%)	60,070	3,931	6.5% (6.3%-6.7%)
**Stem cell transplantation**	10	<5*	*-	18	<5*	*-
**HIV infection**	832	88	10.6% (8.6%-12.7%)	1,040	158	15.2% (13.1%-17.4%)
**Chronic renal failure**	35,644	2,262	6.3% (6.1%-6.6%)	64,701	5,714	8.8% (8.6%-9.1%)
**Chronic severe liver disease**	7,311	301	4.1% (3.7%-4.6%)	10,654	580	5.4% (5.0%-5.9%)
**Immunosuppressant use with RA**	2,257	292	12.9% (11.6%-14.3%)	3,258	469	14.4% (13.2%-15.6%)
**Immunosuppressant use without RA**	5,241	368	7.0% (6.3%-7.7%)	7,573	668	8.8% (8.2%-9.5%)
**Chronic disease present during baseline **						
**Yes**	101,668	5,906	5.8% (5.7%-6.0%)	160,139	12,620	7.9% (7.7%-8.0%)
**No**	91,853	2,495	2.7% (2.6%-2.8%)	129,140	4,734	3.7% (3.6%-3.8%)
**Region**						
**Western German states**	144,737	5,415	3.7% (3.6%-3.8%)	220,669	11,098	5.0% (4.9%-5.1%)
**Eastern German states**	48,274	2,974	6.2% (5.9%-6.4%)	68,083	6,236	9.2% (8.9%-9.4%)

RA = Rheumatoid arthritis; CI = confidence intervals:

*since number of vaccinated patients with stem cell transplantation were below 5, numbers are not shown here.

Patients suffering from rheumatoid arthritis using immunosuppressive treatment, those with HIV infection and those having received stem cell transplantation had the highest vaccination rates in both cohorts, with the largest increase over time in vaccination rates (from cohorts A to B) observed in patients with HIV (4.6 percentage points, pp). Vaccination rates were consistently higher in the Eastern German states than Western German states; while vaccination rates increased in both regions over time (cohort A to B), the increase in the Eastern German states was greater.

### Physician specialist administering the pneumococcal vaccine and diagnosing incident disease in immunocompromised patients

In both cohorts, most of the vaccinations were administered by a general practitioner (GP) (93.2% and 94.0% in cohorts A and B respectively), and rarely by other specialists (Table 4). The first diagnosis of the immunocompromised condition was most often made by a GP in both cohorts (Table 5), without any substantial differences between cohorts A and B. Patients in whom the immunocompromised condition was diagnosed by rheumatologists and pneumologists had the highest vaccination rates, while the lowest vaccination rate in immunocompromised patients was observed in patients in which the condition was diagnosed by a pediatrician (see Table 5).

**Table 4:** Physician administering the vaccination in each cohort.

	Overall	
	Cohort A n (%)	Cohort B n (%)
**Total number of subjects**	8,401 (100.0%)	17,354 (100.0%)
**GP**	7,827 (93.2%)	16,310 (94.0%)
**Rheumatologist**	64 (0.8%)	129 (0.7%)
**Oncologist**	16 (0.2%)	32 (0.2%)
**Pneumologist**	153 (1.8%)	308 (1.8%)
**Pediatrician**	60 (0.7%)	105 (0.6%)
**Internist**	78 (0.9%)	143 (0.8%)
**other**	200 (2.4%)	306 (1.8%)
**unknown**	<5 (0.0%)	21 (0.1%)

**Table 5:** Vaccination rates (with 95% CI) by physician specialty first diagnosing the incident immunocompromised condition putting patient at high-risk for pneumococcal disease.

		Overall		
First diagnosis of condition resulting in immunocompromised status made by:	Cohort	N cohort (%)	N vaccinated	Vaccination rates (95% CI)
**GP**	A	82,840 (42.8%)	3,727	4.5% (4.4%-4.6%)
	B	124,277 (43.0%)	7,892	6.4% (6.2%-6.5%)
**Rheumatologist**	A	2,358 (1.2%)	257	10.9% (9.7%-12.2%)
	B	3,669 (1.3%)	461	12.6% (11.5%-13.7%)
**Oncologist**	A	1,910 (1.0%)	86	4.5% (3.6%-5.5%)
	B	2,981 (1.0%)	192	6.4% (5.6%-7.3%)
**Pneumologist**	A	929 (0.5%)	93	10.0% (8.2%-12.0%)
	B	1,326 (0.5%)	166	12.5% (10.8%-14.3%)
**Pediatrician**	A	6,437 (3.3%)	61	0.9% (0.7%-1.2%)
	B	7,737 (2.7%)	71	0.9% (0.7%-1.1%)
**Internist**	A	3,475 (1.8%)	216	6.2% (5.4%-7.0%)
	B	4,833 (1.7%)	381	7.9% (7.1%-8.7%)
**Other**	A	48,403 (25.0%)	1,824	3.8% (3.6%-3.9%)
	B	68,122 (23.5%)	3,573	5.2% (5.1%-5.4%)
**In hospital**	A	44,086 (22.8%)	2,219	5.0% (4.8%-5.2%)
	B	72,327 (25.0%)	4,722	6.5% (6.3%-6.7%)
**Unknown**	A	14,616 (7.6%)	775	5.3% (4.9%-5.7%)
	B	21,688 (7.5%)	1,449	6.7% (6.4%-7.0%)

### Cumulative pneumococcal vaccination rate within two years after index date

**Fig 2:**
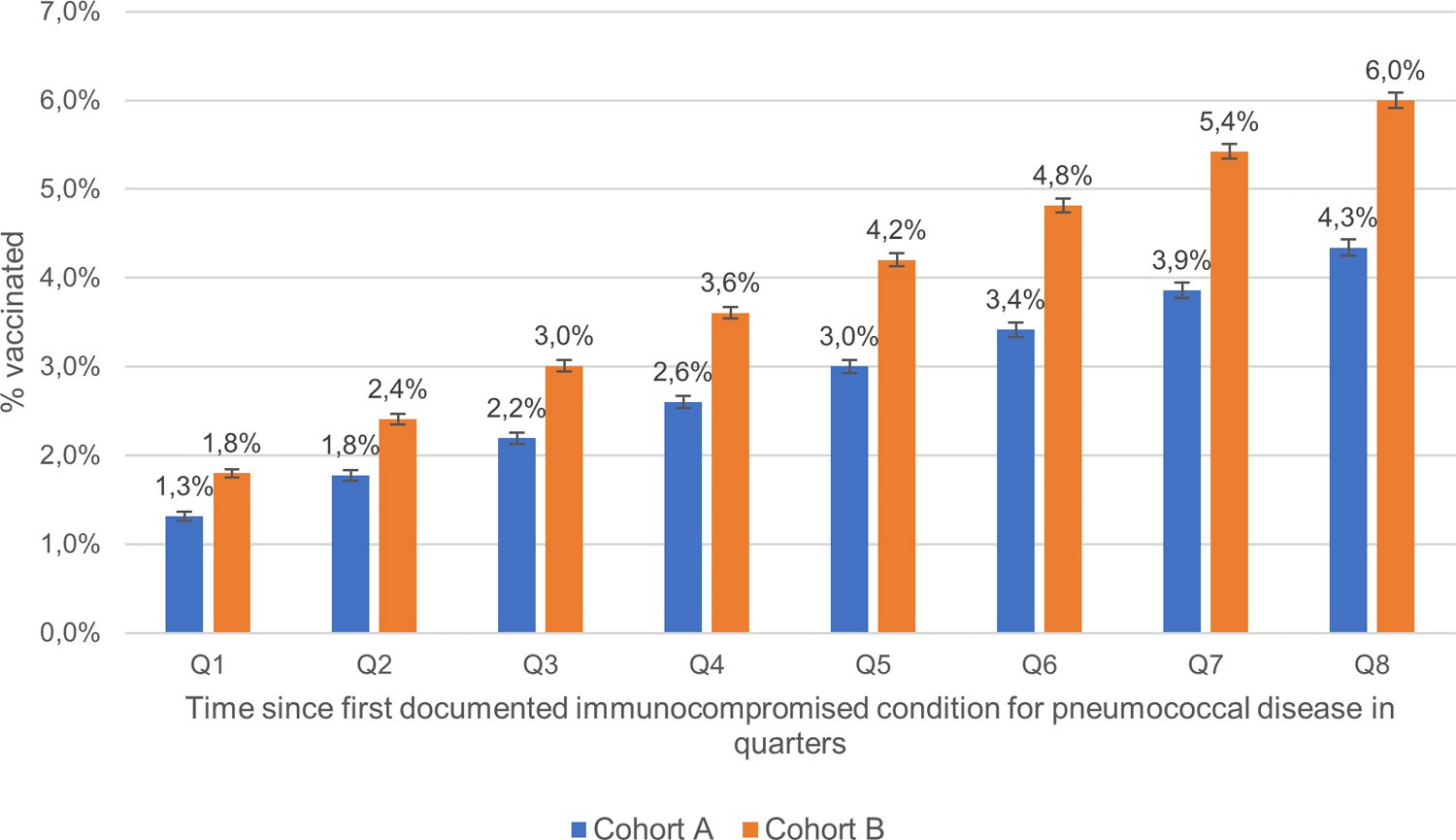
Cumulative pneumococcal vaccination rate within eight quarters after first documented immunocompromised condition by cohort.

The cumulative pneumococcal vaccination rates observed in cohort B were higher at each timepoint (quarter) after diagnosis when compared with cohort A patients (Fig 2). Thus, uptake of vaccination has improved regarding timeliness and vaccination rate between the two cohorts.

### Sequential pneumococcal vaccination

**Fig 3:**
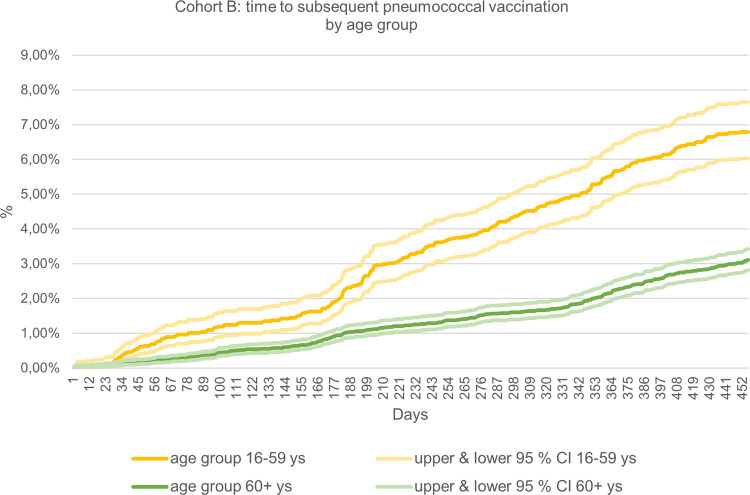
Time (in days) following a first vaccination to a sequential in a 15-month period in cohort B. Patients aged 16–59 (upper and lower 95% CI) compared to patients aged ≥60 years (upper and lower 95% CI) patients (see S4 Table in supplemental material for 95% CIs).

As of 2016, STIKO recommends a sequential pneumococcal vaccination for immunocompromised patients [9]. The overall rate of a subsequent (sequential) vaccination 15 months after an initial pneumococcal vaccination in cohort B was 4.03% (3.74%-4.34%) (Table 6). We see the vaccination rates appear to increase faster around 180 days following the first vaccination in the age group 16–59, just around the 6-month period (Fig 3).

**Table 6:** % of patients with a sequential vaccination following a first vaccination to a sequential vaccination in a 15-month period in cohort B patients aged 16–59 (upper and lower 95% CI) and ≥60 years (upper and lower 95% CI).

	Overall			Age group 16–59			Age group ≥60		
	%	lower 95% CI	upper 95% CI	%	lower 95% CI	upper 95% CI	%	lower 95% CI	upper 95% CI
**15 months (456 days)**	4.03	3.74	4.34	6.79	6.03	7.65	3.11	2.82	3.43

## Discussion

Our study shows that pneumococcal vaccination rates in newly diagnosed immunocompromised patients rose over time and in all observed subgroups: the vaccination rate in cohort A (incident diagnosis in 2013 or 2014) was 4.3%, whereas the rate increased to 6.0% in cohort B (incident diagnosis during 2015 to 2017). The highest vaccination rates were seen among adult patients ≥60 years; men had slightly higher rates in each age group than women and rates were higher in the Eastern than in the Western German States. The lowest vaccination rates were seen in patients aged 2–15 years with less than 1.0% in both cohorts. One possible reason for this may be that no need for further vaccination is seen in this age group, as there is already a vaccination recommendation for infants aged 0–2 years. Vaccination rates in disease-specific subgroups were highest for patients with rheumatoid arthritis using immunosuppressants and for patients with HIV infection; rates were higher for patients with a chronic disease documented during baseline, than for those without. Patients were by far most likely to be vaccinated by their GP, and among the specialists we analysed, pneumologists vaccinated the most patients. When we calculated vaccination rates by the physician specialty that made the diagnosis of the incident immunocompromising condition, we found rheumatologist and pneumologist diagnoses resulted in the highest vaccination rates. The sequential vaccination rate was very low. It was highest at all timepoints in patients aged 16–59 years, suggesting that perhaps immunocompromised patients ≥60 years of age may have received the standard vaccination schedule for healthy individuals of their age group, which does not require a second vaccination within 6–12 months after the first.

Applying the same methodology as described by Schmedt et al. [12] and using a cohort with the same study index period allowed us to compare our findings with their study, to assess trends in time and to evaluate the impact of the updated STIKO recommendations [16] on vaccination rates (including the sequential vaccination scheme). Schmedt et al. [12] used SHI claims data from various sickness funds to observe vaccination rates in incident immunocompromised patients. Patients evaluated in their study had similar demographics to our cohort A; 56.0% of their patients were female, and the overall cumulative vaccination rate over two years was 4.4% (4.3%-4.5%). Our cohort A showed an almost similar vaccination rate of 4.3% (4.3%-4.4%). There are only minor differences in vaccination rates by gender and age as well as region or in the disease-specific subgroups (with partially overlapping 95%-CIs) between the cohort in Schmedt et al. [12] and our study cohort A. Therefore, our approach of comparing two different cohorts in our database to observe trends in time and to evaluate the rate of the sequential vaccination schedule is feasible and cohort A is comparable to the cohort in Schmedt et al. [12]. Our analysis showed that vaccination rates increased significantly from cohort A to cohort B. In the age-groups 16–59 and ≥60 years we saw significant increases. Most of the vaccination rates of different immunocompromised groups were higher in our cohort B patients compared to cohort A and previous studies [12]. However, as our cohort B was slightly older and had a nominally higher percentage of males, this might offer one explanation for higher rates in this cohort. On the other hand, vaccination rates may have increased due to rising awareness both in physicians and patients.

Increases in pneumococcal vaccination rates over time were also reported in the literature. The RKI recently reported increased (from 2015) national pneumococcal vaccination rates of 19.0% in 2020. The highest rate was 30.5% in patients aged 70–79 years in 2020 [10]. Rates increased from one age group to the next, and the most substantial increase was between the age groups 50–59 and 60–69 years, (at least 10 pp) from one year to the next [10]. While our patient population was more selective (incident and immunocompromising conditions; immunocompetent patients with chronic disease were not included), our vaccination rates were substantially lower. Our findings are, however, in line with other studies using SHI claims data. Pneumococcal vaccination rates in 2014 of up to 14.8% were reported in patients aged 60–64 with incident disease [11], with a cumulative vaccination rate after just two years of 7.9% [11, 17]. After three years, the rate was 9.9%, somewhat lower than the 11.1% we found after two years follow-up in our ≥60 years patients in cohort B. Some study design differences, such as including immunocompetent patients with chronic diseases [18], using a one-year index period to determine incidence, or not differentiating between incident and prevalent patients [11], may explain the different vaccination rates.

Only 4.03% of patients (3.74%-4.34%) in our study received a second sequential vaccination within 15 months after the first. This rate was at least twice as high in the 16–59 years age group compared with patients aged ≥60 years at every point during these 15 months. Since the sequential vaccination recommendation was only introduced in August 2016, some of our cohort B patients would have likely been vaccinated according to previous guidelines, resulting in a reduced rate observed. It is also worth noting that over half of our cohort B population had at least one additional chronic disease present during baseline. The recommended pneumococcal vaccination schedule for these patients aged ≥16 is the same as the routine vaccination schedule recommended for patients ≥60 years (a single PPSV23 vaccine) (12), which could further explain the relatively low sequential vaccination rate in this patient group. The low occurrence of the sequential vaccination scheme as recommended for the vulnerable immunocompromised patient population may have been influenced by uncertainty about the correct vaccination schedule. As most vaccinations were administered by a GP (over 90%), and as GPs made only around 40% of immunocompromised diagnoses, uncertainty about the responsibility between GPs and specialists such as internists or oncologists might explain the low rates, too. Electronic medical records can provide a future opportunity of better flag immunocompromised diagnoses and improve the process without losing information. Furthermore, further efforts in training of physicians who are either first diagnosing the patients or administering the vaccine could be helpful.

A study evaluating measures to increase vaccination rates in both immunocompetent and immunocompromised patients in the USA (not restricted to incident as in our population) showed, that the measures they implemented (including notification systems for medical staff) did significantly increase vaccination rates in some groups studied; perhaps an area of research to further explore in Germany [19].

Another reason for low vaccination rates could be a possible limited supply of vaccines during the study period as currently seen and discussed in the COVID-19 pandemic. A potential shortage could lead to implicit prioritisation, i.e. physicians might vaccinate only very specific patient groups. The possible low supply could therefore result in lower vaccination rates. In the current pandemic situation, that did not influence our study, we saw an explicit recommendation of STIKO, i.e. in the case of a shortage only patients in higher age groups or with selected underlying diseases should receive the vaccine [10].

### Strengths and limitations

Our study evaluated pneumococcal vaccination rates according to the most current STIKO recommendations; to our knowledge this is the first analysis of the sequential pneumococcal vaccination in place since 2016. One strength of our study is, that it results from a large and representative database of around 4 million patients, yielding precise estimates with strong external validity of pneumococcal vaccination rates from a real-world setting in different age, gender, and disease groups.

Our study results must be considered against the backdrop of some limitations inherent to claims data studies, as discussed in [20].

- Patients not insured in the SHI or those without continuous baseline or follow-up data, for whatever reason (changed SHI provider, poorly documented data, etc.) within the 2 years each of baseline or follow-up, were not included. Although variations in vaccination rates among different groups (e.g. patients insured in statutory vs. private health insurance) cannot be ruled out, we consider this to be a minor limitation.
- With only a two-year baseline period, left truncation in the first year of each of our cohorts’ baseline periods may have led to an underestimation of pneumococcal vaccination rates, e.g., for patients vaccinated prior to the diagnosis of the immunocompromised condition due perhaps to a chronic condition (which over half of each of our cohorts had).
- The database data does not allow us to differentiate between the PCV13 or PPSV23 vaccine types, however our data are nonetheless suitable for evaluating first vaccination rates following a diagnosis of an immunocompromised condition
- Our cohort B, which we used to evaluate sequential vaccinations, was diagnosed with the immunocompromised condition between 2015 and 2017. The sequential vaccination schedule of PCV13 followed by PPSV23 was only introduced in the guidelines in 2016, so our data likely underestimate the number of patients throughout the 15 months, as opposed to if the guidelines had been established from the beginning of this study index period.
- Nearly a quarter (22.8% and 25.0% in cohorts A and B respectively) of immunocompromised conditions were diagnosed in hospital; inpatient vaccinations, although rare, would not be identifiable in our database, leading to underestimation of vaccination rates. However, to our knowledge they are not part of any of the disease-related groups (DRG) usually reimbursed for inpatient care, and we expect inpatient vaccinations to be low.
- Chronic renal failure and chronic severe liver disease were categorised from at-risk to high-risk conditions in the STIKO recommendations (since 2016) and defined as immunocompromised conditions in our study for both cohorts. Thus, cohort A was not in line with the STIKO recommendations with regard to these diseases and cohort B did not fully reflect guidelines. This might provide partial explanation of lower vaccination rates (around 20% of patients had chronic renal failure and just under 4% had chronic severe liver disease). However, since other studies [11, 17, 21] found no change in vaccination rates in patients with a chronic illness or immunocompromised condition, we expect this impact to be minor.

## Conclusion

Our study results show an increase in pneumococcal vaccination rates in patients first diagnosed as immunocompromised from 2013/2014 to 2015–2017. However, rates of these STIKO-recommended pneumococcal vaccinations in vulnerable patient groups remain very low. Further efforts in training of physicians typically first diagnosing these patients should be made. A further opportunity with flagging of immunocompromised diagnoses in electronic medical records for vaccinations may also help, especially for the relatively large proportion of patients diagnosed by their GP or in hospital. Rates of STIKO-recommended sequential vaccination in our study population were also low; they were higher in patients aged 16–59 years than in patients ≥60 years. In particular immunocompromised patients with at least 60 years of age seem to be vaccinated according to the standard vaccination scheme, which is recommended for healthy individuals in that age group and does not reflect the current STIKO recommendation.

## Supporting information

S1 Fig Time (in days) following a first vaccination to a sequential in a 15-month period in cohort B patients overall (upper and lower 95% CI). (TIF)

S1 Table Conditions in immunocompromised patients, for which pneumococcal vaccination is indicated (adapted from RKI vaccine recommendations). (PDF)

S2 Table States covered by east and west regions, of the regional Association of Statutory Health Insurance Physicians (AHIP) in Germany. (PDF)

S3 Table Vaccination rates (with 95% CI) by physician specialty that first diagnosed immunocompromised condition; differences between former east and western German federal states. (PDF)

S4 Table Percentage (%) of patients with a sequential vaccination in days following a first vaccination to a sequential vaccination in a 15-month period in cohort B patients aged 16–59 (upper and lower 95% CI) and ≥60 (upper and lower 95% CI) (data for Fig 3). (PDF)
