# Supplementary figures and images for: Pneumococcal vaccination rates in immunocompromised patients in Germany: A retrospective cohort study to assess sequential vaccination rates and changes over time
Source: PLoS One. 2022 Mar 22;17(3):e0265433. doi: 10.1371/journal.pone.0265433 (PMC8939779; doi:10.1371/journal.pone.0265433)

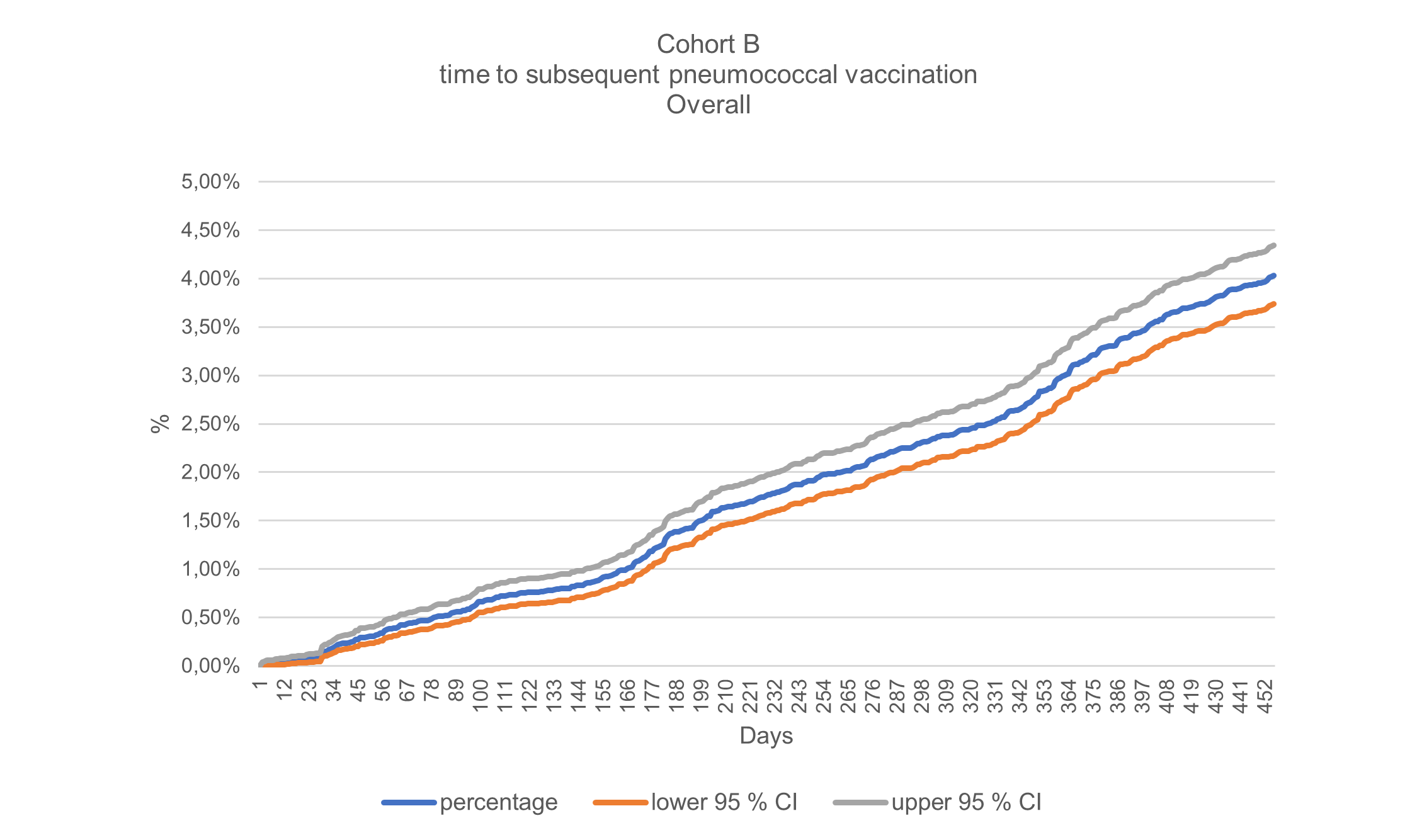

Supplement: S1 Fig — (TIF) [file pone.0265433.s001.tif]
