# Supplementary material for: Pneumococcal vaccination rates in immunocompromised patients in Germany: A retrospective cohort study to assess sequential vaccination rates and changes over time
Source: PLoS One. 2022 Mar 22;17(3):e0265433. doi: 10.1371/journal.pone.0265433 (PMC8939779; doi:10.1371/journal.pone.0265433)
Supplement: S1 Table — (PDF) [file pone.0265433.s002.pdf]

Table S1 Conditions in immunocompromised patients, for which pneumococcal vaccination is indicated (adapted from RKI vaccine recommendations [1]

Vaccination against	Category	Indication	Notes on implementation
**Pneumococcal diseases**	**I**	Children, adolescents and adults with increased health risks due to an underlying condition:<br>1. **Congenital or acquired immunodeficiency and immunosuppression**, eg.:<br>• T-cell deficiency or impaired T-cell function<br>• B-cell or antibody deficiency (eg. hypogammaglobulinemia)<br>• Deficiency or impaired function of myeloid cells (eg. neutropenia, chronic granulomatosis, leucocyte adhesion deficiency, signal transmission defects)<br>• Complement and properdin deficiency<br>• Functional hyposplenism (eg. sickle cell anemia), splenectomy* or anatomical asplenia<br>• Neoplastic diseases<br>• HIV infection<br>• Following bone marrow transplant<br>• Immunosuppressive therapy* (eg. due to organ transplant or autoimmune disease)<br>• Immunodeficiency with chronic kidney failure, nephrotic syndrome or chronic liver failure<br>2. **Other chronic diseases,** eg.:<br>• Chronic cardiac or pulmonary illnesses (eg. asthma, pulmonary emphysema, COPD)<br>• Metabolic illnesses, eg. diabetes mellitus treated with oral medication or insulin.<br>• Neurological illnesses, eg. cerebral palsy or seizures<br>3. **Anatomical and foreign object-associated risks of pneumococcal meningitis,** eg.:<br>• Cerebrospinal fluid fistula<br>• Cochlear implant*<br>____________<br>* vaccination before starting therapy or performing the procedure when possible	1. Sequential vaccination with the 13-valent pneumococcal conjugate vaccine (PCV13) followed by PPSV23 after 6-12 months. The PPSV23 vaccine should only be administered to those aged 2+ **<br>2. In those 16+ to receive 1 dose of PPSV23.** Persons 2-15 years to receive sequential vaccination with PCV13 followed by PPSV23 after 6-12 months.**<br>3. Sequential vaccination with PCV13 followed by PPSV23 after 6-12 months. The PPSV23 vaccine should only be administered to those aged 2+ years.**<br>____________<br>** due to the limited duration of the protection given by the PPSV23 vaccination, PPSV23 vaccination should be repeated after at least 6 years.

References

1. Gemeinsamer Bundesausschuss, Richtlinie des Gemeinsamen Bundesausschusses. über Schutzimpfungen nach § 20i Absatz 1 SGB V. Available from: https://www.g-ba.de/downloads/62-492-2429/SI-RL_2021-03-21_iK-2021-04-01.pdf.
