# Supplementary material for: Pneumococcal vaccination rates in immunocompromised patients in Germany: A retrospective cohort study to assess sequential vaccination rates and changes over time
Source: PLoS One. 2022 Mar 22;17(3):e0265433. doi: 10.1371/journal.pone.0265433 (PMC8939779; doi:10.1371/journal.pone.0265433)
Supplement: S2 Table — (PDF) [file pone.0265433.s003.pdf]

Table S2 States covered by east and west regions, of the regional Association of Statutory Health Insurance Physicians (AHIP) in Germany

Eastern German States	Western German states
• Berlin<br>• Mecklenburg-Western Pomerania<br>• Brandenburg<br>• Saxony-Anhalt<br>• Thuringia<br>• Saxony	• Schleswig-Holstein<br>• Hamburg<br>• Bremen<br>• Lower Saxony<br>• Westfalia-Lippe<br>• Northrine<br>• Hesse<br>• Rhineland-Palatinate<br>• Baden Wuerttemberg<br>• Bavaria<br>• Saarland
