# Supplementary material for: Pneumococcal vaccination rates in immunocompromised patients in Germany: A retrospective cohort study to assess sequential vaccination rates and changes over time
Source: PLoS One. 2022 Mar 22;17(3):e0265433. doi: 10.1371/journal.pone.0265433 (PMC8939779; doi:10.1371/journal.pone.0265433)
Supplement: S3 Table — (PDF) [file pone.0265433.s004.pdf]

Table S3 Vaccination rates (with 95% CI) by physician specialty that first diagnosed immunocompromised condition; differences between former east and western German federal states

		Germany (former) West (without Berlin)			Germany (former) East (with Berlin)		
First diagnosis of condition resulting in immunocompromised status:	Cohort	N cohort (%)	N vaccinated	Vaccination rates (95% CI)	N cohort (%)	N vaccinated	Vaccination rates (95% CI)
GP	A	63,560 (43.9%)	2,434	3.8% (3.7%-4.0%)	19,011 (39.4%)	1,286	6.8% (6.4%-7.1%)
	B	96,383 (43.7%)	5,069	5.3% (5.1%-5.4%)	27,648 (40.6%)	2,815	10.2% (9.8%-10.5%)
Rheumatologist	A	1,668 (1.2%)	163	9.8% (8.4%-11.2%)	689 (1.4%)	94	13.6% (11.2%-16.3%)
	B	2,633 (1.2%)	299	11.4% (10.2%-12.6%)	1,035 (1.5%)	162	15.7% (13.5%-17.9%)
Oncologist	A	1,339 (0.9%)	41	3.1% (2.2%-4.0%)	569 (1.2%)	45	7.9% (5.8%-10.2%)
	B	2,282 (1.0%)	130	5.7% (4.8%-6.7%)	687 (1.0%)	61	8.9% (6.9%-11.1%)
Pneumologist	A	691 (0.5%)	67	9.7% (7.6%-12.0%)	233 (0.5%)	26	11.2% (7.4%-15.4%)
	B	1,037 (0.5%)	106	10.2% (8.4%-12.1%)	287 (0.4%)	60	20.9% (16.3%-25.7%)
Pediatrician	A	4,887 (3.4%)	36	0.7% (0.5%-1.0%)	1,531 (3.2%)	25	1.6% (1.1%-2.3%)
	B	5,908 (2.7%)	51	0.9% (0.6%-1.1%)	1,818 (2.7%)	20	1.1% (0.7%-1.6%)
Internist	A	2,312 (1.6%)	138	6.0% (5.0%-7.0%)	1,156 (2.4%)	78	6.7% (5.4%-8.3%)
	B	3,486 (1.6%)	246	7.1% (6.2%-7.9%)	1,340 (2.0%)	135	10.1% (8.5%-11.7%)
Other	A	34,984 (24.2%)	1,118	3.2% (3.0%-3.4%)	13,316 (27.6%)	704	5.3% (4.9%-5.7%)
	B	51,123 (23.2%)	2,204	4.3% (4.1%-4.5%)	16,870 (24.8%)	1,363	8.1% (7.7%-8.5%)
In hospital	A	31,920 (22.1%)	1,462	4.6% (4.4%-4.8%)	12,071 (25.0%)	754	6.2% (5.8%-6.7%)
	B	53,621 (24.3%)	3,030	5.7% (5.5%-5.8%)	18,589 (27.3%)	1,686	9.1% (8.7%-9.5%)
Unknown	A	11,843 (8.2%)	487	4.1% (3.8%-4.5%)	2741 (5.7%)	288	10.5% (9.4%-11.7%)
	B	17,309 (7.8%)	951	5.5% (5.2%-5.8%)	4356 (6.4%)	498	11.4% (10.5%-12.4%)
