# Supplementary material for: Pneumococcal vaccination rates in immunocompromised patients in Germany: A retrospective cohort study to assess sequential vaccination rates and changes over time
Source: PLoS One. 2022 Mar 22;17(3):e0265433. doi: 10.1371/journal.pone.0265433 (PMC8939779; doi:10.1371/journal.pone.0265433)
Supplement: S4 Table — (PDF) [file pone.0265433.s005.pdf]

Table S4: Percentage (%) of patients with a sequential vaccination in days following a first vaccination to a sequential vaccination in a 15-month period in cohort B patients aged 16-59 ys (upper and lower 95% CI) and ≥60 ys (upper and lower 95% CI) (data for Fig 3)

	Age group 16-59 ys			Age group ≥60 ys		
time to second vaccination (days)	%	lower 95% CI	upper 95% CI	%	lower 95% CI	upper 95% CI
1	0.00%	0.00%	0.00%	0.00%	0.00%	0.00%
15	0.08%	0.02%	0.23%	0.05%	0.02%	0.10%
29	0.20%	0.10%	0.40%	0.10%	0.06%	0.17%
43	0.53%	0.35%	0.81%	0.14%	0.09%	0.22%
57	0.73%	0.51%	1.05%	0.21%	0.15%	0.31%
71	0.94%	0.68%	1.29%	0.26%	0.18%	0.36%
85	1.01%	0.74%	1.38%	0.32%	0.24%	0.43%
99	1.19%	0.90%	1.58%	0.44%	0.34%	0.57%
113	1.29%	0.98%	1.70%	0.51%	0.40%	0.65%
127	1.29%	0.98%	1.70%	0.55%	0.43%	0.69%
141	1.42%	1.10%	1.84%	0.58%	0.46%	0.72%
155	1.55%	1.21%	1.99%	0.66%	0.54%	0.82%
169	1.71%	1.35%	2.16%	0.80%	0.66%	0.97%
183	2.18%	1.76%	2.69%	0.98%	0.82%	1.16%
197	2.62%	2.16%	3.17%	1.07%	0.91%	1.26%
211	2.99%	2.50%	3.57%	1.17%	1.00%	1.37%
225	3.17%	2.66%	3.77%	1.23%	1.05%	1.43%
239	3.49%	2.95%	4.11%	1.27%	1.10%	1.48%
253	3.67%	3.12%	4.31%	1.37%	1.18%	1.58%
267	3.78%	3.22%	4.43%	1.42%	1.23%	1.64%
281	3.96%	3.39%	4.63%	1.55%	1.35%	1.78%
295	4.26%	3.66%	4.94%	1.59%	1.39%	1.82%
309	4.52%	3.91%	5.23%	1.63%	1.43%	1.86%
323	4.77%	4.14%	5.49%	1.69%	1.49%	1.93%
337	4.95%	4.31%	5.69%	1.82%	1.61%	2.07%
351	5.28%	4.62%	6.04%	1.99%	1.77%	2.25%
365	5.61%	4.92%	6.39%	2.22%	1.98%	2.49%
379	5.91%	5.21%	6.71%	2.39%	2.14%	2.67%
393	6.05%	5.34%	6.86%	2.55%	2.29%	2.84%
407	6.30%	5.57%	7.12%	2.72%	2.45%	3.01%
421	6.50%	5.76%	7.34%	2.80%	2.53%	3.10%
435	6.71%	5.95%	7.55%	2.91%	2.63%	3.22%
449	6.77%	6.01%	7.62%	3.02%	2.73%	3.33%
